# Is the behavioural divergence between range-core and range-edge populations of cane toads (*Rhinella marina*) due to evolutionary change or developmental plasticity?

**DOI:** 10.1098/rsos.170789

**Published:** 2017-10-25

**Authors:** Jodie Gruber, Gregory Brown, Martin J. Whiting, Richard Shine

**Affiliations:** 1 School of Life and Environmental Sciences, The University of Sydney, Sydney, New South Wales, Australia; 2 Department of Biological Sciences, Macquarie University, North Ryde, New South Wales, Australia

**Keywords:** adaptation, *Bufo marinus*, evolution, spatial sorting

## Abstract

Individuals at the leading edge of expanding biological invasions often show distinctive phenotypic traits, in ways that enhance their ability to disperse rapidly and to function effectively in novel environments. Cane toads (*Rhinella marina*) at the invasion front in Australia exhibit shifts in morphology, physiology and behaviour (directionality of dispersal, boldness, risk-taking). We took a common-garden approach, raising toads from range-core and range-edge populations in captivity, to see if the behavioural divergences observed in wild-caught toads are also evident in common-garden offspring. Captive-raised toads from the invasion vanguard population were more exploratory and bolder (more prone to ‘risky’ behaviours) than toads from the range core, which suggests that these are evolved, genetic traits. Our study highlights the importance of behaviour as being potentially adaptive in invasive populations and adds these behavioural traits to the increasing list of phenotypic traits that have evolved rapidly during the toads' 80-year spread through tropical Australia.

## 1. Introduction

Biological invasions impose novel ecological and evolutionary pressures. Individuals at the expanding range edge experience different demographic, physiological and environmental pressures than those faced by conspecifics from the range core, often leading to phenotypic divergence across the invasion range [1]. For example, vanguard individuals often have distinctive phenotypes associated with faster dispersal such as larger size [2–4], longer legs [5] and increased investment in dispersal appendages and mass in plant seeds [6–8]. More generally, successful invaders exhibit a suite of physiological, life-history, morphological and behavioural traits that enhance dispersal rates and facilitate functioning in novel environments (known as an ‘Invasion Syndrome’; [9,10]). Behavioural traits may play an especially critical role (both as direct facilitators of dispersal rate, and via their influence on selection pressures on other traits), and yet are often overlooked as factors contributing to invasion success [10,11].

A propensity to explore, take risks and engage with novel environments (neophilia) is likely to promote range expansion by stimulating dispersal [12,13], and these traits also enhance an individual's ability to find water, food, shelter and mates in novel environments [11,14,15]. In keeping with these predictions, behavioural traits have been linked to range expansion and invasion success in several species. For example, range-edge individuals are more exploratory and bolder (more willing to take risks) than are conspecifics from range-core populations of the round goby (*Neogobius melanostomus*) [16], dark-eyed junco (*Junco hyemalis Thurber*) [17,18], common frog (*Rana temporaria*) [19] and house sparrow (*Passer domesticus*) [20]. Invasive house sparrows from range-edge populations are also more neophilic than are conspecifics from range-core populations [21,22]. Individuals from range-edge populations also may be more aggressive [23,24] and less social [3,25] compared with those from range-core populations.

Despite the growing body of evidence for distinctive behavioural phenotypes at range edges, the causation for this pattern is ambiguous [1]. The two broad categories of explanation involve (i) behavioural plasticity, induced by the novel environments experienced at the range edge and (ii) the rapid elaboration of heritable traits driven by the unique evolutionary pressures imposed by an invasion.

Many behavioural phenotypes are highly labile, enabling organisms to plastically respond to changes in their physiological, climatic or social environments [26]. Thus, range-edge individuals may shift their behaviour in response to the novel environmental pressures they encounter at the expanding range edge [27,28]. An ability to rapidly shift behaviour in response to novelty may enhance an individual's ability to exploit new ecological niches, habitats or prey items at the range edge [29]. Indeed, innovation has been linked to invasion success in several species [30] including the black rat (*Rattus rattus*) [31] and bird species worldwide [30,32]. In summary, range-edge individuals may exhibit specific behavioural attributes because those attributes are induced by the novel (range-edge) conditions that an individual experiences during their life.

Alternatively, the distinctive behaviour of invasion-front animals may reflect rapid evolutionary change. Behavioural traits are affected by genes [33], and traits such as exploration and boldness [34–36] and aggression [37] are heritable in some species (reviewed in [38,39]). Variation in behavioural phenotypes between range-core versus range-edge populations is likely to have both ecological and evolutionary consequences through influences on life-history, physiological and morphological traits, and hence be a target for selection [27]. For example, dispersing individuals at the range edge may benefit from reduced competition for resources, a lack of pathogens and parasites, access to the best habitat and a lower risk of predation [40,41]. However, there are also potential costs associated with rapid dispersal, such as the energetic costs of activity, increased risk of injury and a decrease in opportunities to reproduce due to low densities at the range edge [42]. Hence, the benefits of dispersal must outweigh the costs for dispersal-enhancing behavioural traits to be adaptive. Importantly, though, personality-dependent dispersal may evolve even in the absence of natural selection. Bold and neophilic individuals are likely to be the first to disperse out of the range core, where they interbreed. At least some of their progeny inherit dispersal-enhancing traits from both parents, leading to a progressive increase across generations in the dispersal rates of individuals at the range edge (dubbed ‘spatial sorting’ by Shine *et al*. [43]). These proximate mechanisms (phenotypic plasticity, adaptation, spatial sorting) are not mutually exclusive; for example, an invasion might favour the evolution of specific reaction norms.

To investigate the causal factors driving behavioural divergence across a biological invasion, the first step is to disentangle plastic responses to novel environments from heritable shifts in behaviour. To answer this question, we need to measure behavioural traits not only of wild-caught individuals, but also of individuals raised in standardized (common-garden) conditions. In this study, we gathered these two types of data on the cane toad (*Rhinella marina* Linnaeus 1758) and its ongoing invasion across Australia.

The cane toad's current invasion range extends from wet tropical Queensland in the northeast to the seasonally dry monsoonal climate of northwestern Western Australia [44]. Hence, toads from the range front versus the range edge experience very different thermal and hydric regimes; environmental factors which can have strong effects on the development of amphibians [45–47]. Previous research has documented rapid evolution of dispersal-enhancing morphological [48], physiological [49] and life-history traits [5,41,48,50–52] across this invasion range. Many of the same traits are seen in offspring raised in ‘common-garden’ experiments, suggesting a heritable component [50,53,54]. Invasion-front toads also are more exploratory and willing to take risks than are toads from long-colonized populations [55], but the heritability of these latter traits has not been investigated using common-garden methods to date. The cane toad's invasion of Australia provides an ideal model system in which to ask our key question: is behavioural divergence across the invasion range due to plastic responses to the different climatic, physical and demographic environments experienced by range-front versus range-core individuals, or are they due to rapid evolutionary (heritable) changes? To answer this question, we conducted standardized laboratory-based behavioural assays to quantify exploratory, risk-taking and neophilic behaviour in both wild-caught and captive-raised cane toads from the range core and range edge of their Australian invasion range.

## 2. Material and methods

### 2.1. Collection of wild-caught toads

In 2016, we collected 68 adult cane toads (34 male, 34 female), half from a long-colonized population (Cairns, Queensland: 17°56′ S, 145°56′ E; more than 76 years post-colonization [51]; mean annual rainfall: 1999.7 mm, mean annual maximum temperature: 29.0°) and half from a range-front population (Oombulgurri, Western Australia: 15°10′45′′ S,127°52′36′′ E; less than 3 years post-colonization [56]; mean annual rainfall: 809.4 mm, mean annual maximum temperature: 35.1° for Kununurra; Australian Government Bureau of Meteorology (www.bom.gov.au) 2016). Toads were collected from three sub-populations within each location, hence the years since colonization (above) are means for each area. Our collection and transportation procedures are described in Gruber *et al*. [55]. Toads were kept at the University of Sydney's Tropical Ecology Research Facility at Middle Point, Northern Territory (12°34′ S, 131°18′ E; 11 years post-colonization; mean annual rainfall: 1421.7 mm, mean annual maximum temperature: 33.1°; Australian Government Bureau of Meteorology (www.bom.gov.au) 2016). Upon collection, toads were weighed to the nearest 0.1 g on a digital scale and measured (snout–urostyle length (SUL)) to the nearest 0.01 mm using digital callipers.

### 2.2. Rearing of toads in captivity

Our common-garden toads were bred in captivity from wild-caught Western Australian and Queensland toads (for collection details and breeding protocols, see [57,58]). All dams and sires were bred only once, hence all F_1_ individuals from each clutch were full-siblings [58]. We used individuals from 13 clutches from Western Australian and 16 clutches from Queensland ancestry. At the time of testing, these captive-raised toads were approximately 18 months of age and had been raised for their entire lives under standard conditions at the same site (our Northern Territory research station, as described above). All toads were weighed and measured before being housed as described below.

### 2.3. Methods for general husbandry

At the research station, toads were transferred from their large outdoor pens [58] to smaller containers (100 l plastic tubs with mesh lids) where they were housed in groups of two or three. Each tub had a wood-shaving substrate and contained two shelters (each shelter was large enough to hold three toads) and a large shallow water dish. Toads were fed live crickets four times per week and were provided with fresh water ad libitum. Toads were weighed and measured regularly to monitor their health (average toad SUL 98 mm; average toad mass 120 g). None showed any signs of illness or weight loss. Handling of toads was kept to a minimum and, to reduce stress, toads were transferred to and from trial arenas in dark plastic tubs. To minimize stress, toads were left undisturbed during non-trial periods.

### 2.4. Trial methods

As adult toads are most active at night [59,60], we conducted behavioural trials between 18.30 and 01.00 h. Each toad was tested in three different behavioural trials: (i) exploration of a novel arena, (ii) risk-taking (emergence from a shelter into a test arena), and (iii) neophilia (response to a novel object). Trials ran for 30 min and four trials with one toad per trial arena were conducted simultaneously in each trial round. Trials were split over 3 days with six trial rounds on the first two nights (24 toads tested on each night) and five (20 toads tested) on the third night. All toads were exposed to each behavioural trial in the same sequence, that is, an exploratory trial, a risk-taking trial and a neophilia trial with 2 days of rest in between trial types (while the other sets of toads were assayed). An equal number of toads from range-front and range-core populations and each sex were randomly allocated a trial time and arena within each trial day. Toads from the common-garden and wild-caught populations were assayed consecutively rather than simultaneously due to logistical and space constraints.

Trial arenas were large (120 × 120 × 83 cm) hexagonal pens made from waterproof fabric with an open top to allow filming from above. The PVC substrate (and shelters, etc.) of each arena were wiped with diluted ethanol before each trial to eliminate scent. We measured the arena floor temperature before each trial (range: 29–31°C). We included an empty container in all trials to disambiguate hiding behaviour from interest in a novel object (for consistency, empty containers were also included in exploration and emergence trials). At the start of each exploratory and neophilia trial, toads were given 5 min before their resting shelter was removed and trials began. During risk-taking trials, toads began the trial in the safety of the shelter and the entrance was covered for five minutes before the cover was removed. All trials were recorded using CCTV cameras and we scored videos using Ethovision XT10 behavioural analysis software. Ethovision scored all videos in a standardized way without information on the population of origin (to ensure blind scoring). The investigator left the room during trials to avoid affecting toad behaviour.

#### 2.4.1. Exploratory trial

To test exploration and space use in a novel environment [3,13], we measured the time spent moving and rate of movement [61,62]. We provided a shelter in the arena to give toads the option to hide during trials. This allowed us to distinguish bold exploratory behaviour from fear-driven escape behaviour [63]. An empty container was placed next to the shelter and both were positioned equidistant to one another at the opposite end of the arena from the start point.

#### 2.4.2. Emergence trial

To score risk-taking behaviour, we recorded whether or not a toad emerged from its shelter, and latency to emerge (s). Emergence during the trial and a shorter latency to emerge indicates higher risk-taking propensity [3,19]. Two empty containers were placed at the opposite end of the arena from the start point.

#### 2.4.3. Novel object trial

To test neophilia, we used a silicone fishing lure (20 mm × 10 mm, mimicking a squid) driven by a small motor to move up and down every 2 s as a novel object. The novel object was housed inside a clear container to prevent the toad from consuming the plastic lure. The novel object was placed at the opposite end of the arena to the start point.

### 2.5. Statistical analysis

We used linear models to analyse the effects of population source (Western Australia–Queensland), population type (common-garden–wild-caught) and population source within population type separately (common-garden toads from Western Australian versus Queensland ancestry and wild-caught toads from Western Australia and Queensland) on behavioural traits. We did not incorporate clutch as a factor in our analysis as we were not able to obtain information on relatedness in wild toads. The specific measurements collected from each trial were as follows:

(1) exploratory trials—total time spent moving and rate of movement (as quantified by the residual scores from a simple linear regression of total distance moved against total time spent moving);
(2) emergence trials—emergence (binomial, whether or not individuals emerged during trials) and latency to emerge; and
(3) novel object trials—whether or not toads approached to within 90 mm (the minimum body length required for toads to be classed as adults [64]; thus, toads were recorded as being in the ‘novel object zone’ when within one body length of the focal object) of the novel object (binomial), latency to approach and time spent within 90 mm of the novel object.

Repeatabilities of these behaviours (obtained from a larger sample of toads subjected to these trials on two occasions) ranged from *R* = 0.01 (±95% CI 0, 0.56) *p* = 0.50 for activity to *R* = 0.49 (±95% CI 0.31, 0.81) *p* = 0.001 for latency to emerge (Gruber *et al*. 2017, unpublished data). We included data from captive-raised and wild-caught toads in a single model to examine the effects of population (range-core and range-edge), source (captive-raised and wild-caught) and their interaction on toad behaviour. We used top-down stepwise model selection, starting with a full model including all factors, covariates and their interactions, and sequentially deleted non-significant terms. Sex, mass, arena temperature, arena number, time of trial and day of trial had non-significant effects on behavioural traits and thus were excluded from the final models. We did not detect significant interactions between any factors, hence all interaction terms were also removed from the final models.

## 3. Results

Overall, toads from the range edge were more exploratory and had a higher propensity to take risks than did toads from the range core (table 1). We found no significant interaction effect between population (range-edge versus range-core) and prior experience (captive-raised versus wild-caught) on any behavioural traits. Range-edge and range-core toads exhibited similar rates of movement during exploration trials, were similarly likely to emerge during risk-taking trials and responded in similar ways to the novel object during neophilia trials (table 1).

**Table 1.** Effects of population (range-core versus range-edge), source (captive-raised versus wild-caught) and their interaction (population × source) on behavioural traits during exploration (time spent moving, rate of movement), risk-taking (proportion to emerge and latency to emerge) and neophilia (proportion to approach novel object and time spent with novel object) trials. Results for main effects are based on analyses after exclusion of non-significant interaction terms. Statistically significant values (*p* < 0.05) are highlighted in bold text.

variable	population	captive-raised versus wild	population × source
time spent moving (s)	***F*_1,135_ = 4.40**	***F*_1,135_ = 35.19**	*F*_1,135_ = 0.45
	***p* < 0.04**	***p* d 0.0001**	*p* = 0.50
movement rate	*F*_1,135_ = 0.42	***F*_1,135_ = 31.43**	*F*_1,135_ = 0.11
	*p* = 0.52	***p* < 0.0001**	*p* = 0.74
proportion to emerge	*χ*^2 ^= 1.57	*χ*^2 ^= 0.00	*χ*^2 ^= 0.03
	*p* = 0.21	*p* = 0.1	*p* = 0.86
emergence latency (s)	***F*_1,135_ = 12.77**	*F*_1,135_ = 0.68	*F*_1,135_ = 0.026
	***p* = 0.0005**	*p* = 0.41	*p* = 0.87
proportion to approach novel object	*χ*^2 ^= 0.08	*χ*^2 ^= 0.16	*χ*^2 ^= 0.16
	*p* = 0.78	*p* = 0.78	*p* = 0.69
time spent with novel object (s)	*F*_1,135_ = 0.17	*F*_1,135_ = 3.79	*F*_1,135_ = 0.06
	*p* = 0.68	*p* = 0.054	*p* = 0.80

**Figure 1.**
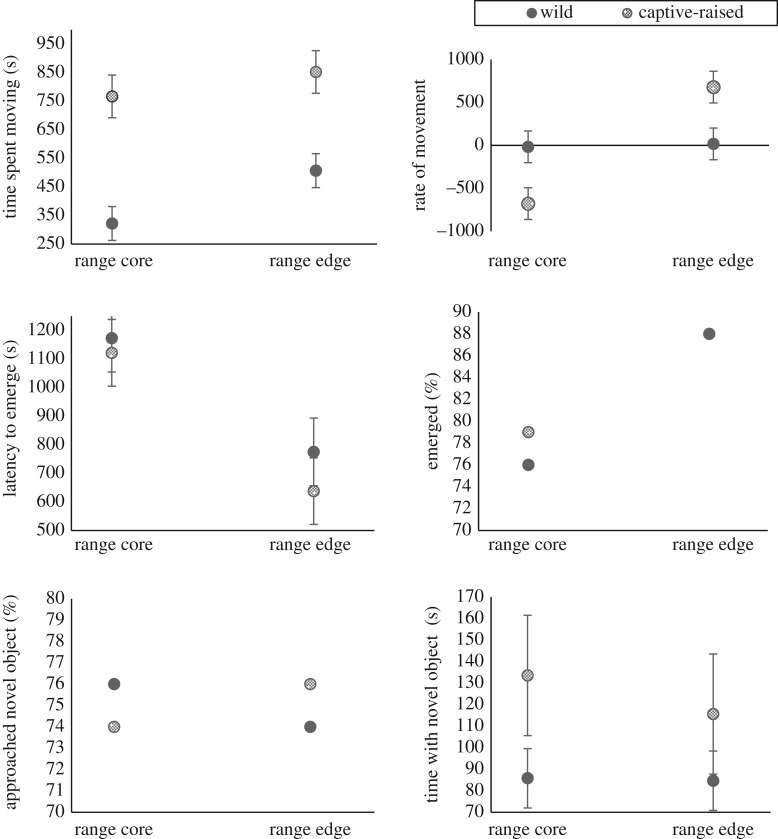
Behavioural traits of cane toads from wild-caught and captive-raised populations of Western Australian (range-edge) and Queensland (range-core) origin. Graphs show mean values and associated standard errors (where relevant) for traits measured during trials of exploratory behaviour (time spent moving, rate of movement), risk-taking (emerged from shelter, latency to emerge) and neophilia (approached novel object, time with novel object).

During exploration trials, captive-raised toads spent more time moving and moved faster than did wild-caught conspecifics (table 1; figure 1). The proportion of toads to emerge during risk-taking trials and to approach the novel object during neophilia trials did not differ between captive-raised versus wild-caught toads (table 1; figure 1). Captive-raised toads were quicker to emerge from the shelter during emergence trials and spent more time with the novel object during novel object trials than did wild-caught toads, but these results did not reach *α *< 0.05 (table 1).

## 4. Discussion

Our findings are consistent with those of previous studies on behavioural divergence across the cane toad's Australian invasion range [55]: range-edge toads were more exploratory and willing to take risks than were conspecifics from range-core populations. Importantly, the divergence in risk-taking behaviour was seen in captive-raised toads as well as wild-caught animals (and was statistically significant in analyses restricted only to data from captive-raised animals). The divergence in risk-taking behaviour in toads from different ancestral populations, even after the animals were raised in identical conditions, indicates a heritable component to this behaviour. Hence, behavioural divergence across the invasion range in this species cannot be due entirely to plastic responses to different environments.

The distinctive behavioural traits seen at expanding range edges may result from both adaptive and non-adaptive processes. First, traits that enhance dispersal and adaptation to novel environments may confer fitness advantages at the range edge, and hence evolve via natural selection [16]. For example, ‘risk-taking’ individuals may be more likely to disperse into novel environments where competition for resources (such as food and shelter) and the numbers of pathogens and parasites are reduced by low densities of conspecifics [16,17,41,65]. High levels of exploration, risk-taking and neophilia also may enable individuals to exploit novel niches and resources [21,66,67]. Even in the absence of such adaptive advantages, however, distinctive behavioural phenotypes may accumulate at the invasion front because of spatial sorting [43]. That is, the increasingly fast-moving invasion front inevitably is dominated by fast-dispersing individuals, whose interbreeding produces even faster-dispersing offspring [5,43,68]. The evolution of behavioural traits across an invasion range may also be indirectly affected by the rapid evolution of morphological, physiological or life-history traits if there are genetic links between behaviour and these traits (e.g. the ‘Pace of Life Syndrome’ hypothesis [69]). Other processes such as genetic drift, surfing of deleterious mutations [70,71] and density-dependent selection [57] may also influence the frequency of inherited traits at the invasion front.

Previous research using captive-raised (common-garden) cane toads has documented significant heritability for traits such as dispersal rate [57], limb morphology [58] and locomotor performance [72]. Similarly, heritability of dispersal-related behavioural traits has been documented in the great tit (*Parus major*) [34,65,73,74], the red-cockaded woodpecker (*Picoides borealis*) [75], the collared flycatcher (*Ficedula albicollis*) [76] and the Glanville fritillary butterfly (*Melitaea cinxia*) [77]. Our results on cane toads suggest a heritable component to behavioural traits that enhance dispersal, but we cannot quantify heritability without breeding experiments to allow cross-generational comparisons of behavioural traits between parents and offspring.

Heritability and plasticity are not mutually exclusive mechanisms [78]. Indeed, behavioural plasticity is driven both by environmental conditions and by the constraints of evolved (genetically based) norms of reaction [66]. However, these two responses are closely related, with reaction norms evolving such as to generate the most appropriate response to any given set of environmental conditions [79]. Behavioural plasticity often may be beneficial in a novel environment, if it induces an individual to alter its behaviour appropriately and at a rate that fits the prevailing environmental conditions [27,29,80].

Adaptive activational plasticity occurs when an animal has the ability to express a trait (such as neophilia) but only does so in response to the appropriate stimulus [66]. Activational plasticity may explain why neophilia varied so little among toads in this study (in wild-caught as well as captive-bred populations, and from the range edge as well as the range core). The potential advantage of neophilia at the range edge is clear, as it promotes movement through novel areas and exploitation of novel resources [11,28,67]. Although cane toads from the range core are in a familiar environment, they nonetheless must often encounter novelty (especially, in urban habitats) and neophilic traits may also enhance fitness in this situation. If so, range-core individuals may possess neophilic traits but not express them unless they are exposed to novelty (e.g. during the neophilia trial) [1].

In this study, we investigated whether the behavioural divergence documented in cane toads across their Australian invasion range [55] is solely due to plastic responses to the profoundly different climatic, physical and demographic environments experienced by range-front versus range-core individuals, or if other mechanisms have a role to play. In keeping with the earlier report, we found that range-edge toads were more exploratory and willing to take risks than were toads from long-colonized areas—and importantly, that divergence was evident even if the toads had been raised in captivity. We thus infer that at least part of the behavioural divergence between range-core and range-edge cane toads in Australia is a result of rapid evolutionary change (i.e. heritable factors, that differ among populations), as has been documented also for a wide range of morphological and physiological traits [5,41,48,50–52]. Further work is required to measure heritability in behavioural traits and to determine whether the shifts in these traits during the course of the toads' Australian invasion have been driven by adaptive or non-adaptive processes.

## Supplementary Material

Gruber et al. 2017 Data of behavioural measures from captive-raised and wild-caught cane toads from exploration, risk-taking and neophilia trials

## Data accessibility

The dataset supporting this article has been uploaded as part of the electronic supplementary material.
